# Brain Circuitries Involved in Semantic Interference by Demands of Emotional and Non-Emotional Distractors

**DOI:** 10.1371/journal.pone.0038155

**Published:** 2012-05-29

**Authors:** Natalia Chechko, Thilo Kellermann, Michael Zvyagintsev, Marc Augustin, Frank Schneider, Ute Habel

**Affiliations:** 1 Department of Psychiatry, Psychotherapy and Psychosomatics, Medical School, RWTH Aachen University, Aachen, Germany; 2 JARA–Translational Brain Medicine, Forschungszentrum Jülich GmbH, Jülich, Germany; University of Groningen, Netherlands

## Abstract

**Background:**

Previous studies have indicated that the processes leading to the resolution of emotional and non-emotional interference conflicts are unrelated, involving separate networks. It is also known that conflict resolution itself suggests a considerable overlap of the networks. Our study is an attempt to examine how these findings may be related.

**Methodology/Principal Findings:**

We used functional magnetic resonance imaging (fMRI) to study neural responses of 24 healthy subjects to emotional and non-emotional conflict paradigms involving the presentation of congruent and incongruent word-face pairs based on semantic incompatibility between targets and distractors. In the emotional task, the behavioral interference conflict was greater (compared to the non-emotional task) and was paralleled by involvement of the extrastriate visual and posterodorsal medial frontal cortices. In both tasks, we also observed a common network including the dorsal anterior cingulate, the supplemental motor area, the anterior insula and the inferior prefrontal cortex, indicating that these brain structures are markers of experienced conflict. However, the emotional task involved conflict-triggered networks to a considerably higher degree.

**Conclusions/Significance:**

Our findings indicate that responses to emotional and non-emotional distractors involve the same systems, which are capable of flexible adjustments based on conflict demands. The function of systems related to conflict resolution is likely to be adjusted on the basis of an evaluation process that primarily involves the extrastriate visual cortex, with target playing a significant role.

## Introduction

Goal-directed behavior suggests efficient executive processes enabling one to maintain focus on task-relevant information even while this ability is challenged by potent distractors. Emotional distractors have been proved to be particularly effective in capturing our attention and processing resources. Thus, emotionally significant words divert attention from the main task, making one take longer to name ink colors or count the number of words when the words are emotional compared to when they are neutral [1]–[4]. This effect is also seen in emotional interference tasks such as emotional counting Stroop [2]. Comparing the emotional and non-emotional counting Stroop tasks, a functional segregation of the anterior cingulate cortex (ACC) in cognitive (dorsal) and emotional (rostral) subdivisions has been suggested [2], [5], indicating that the resolution of emotional and non-emotional interference conflicts might entail different processes, involving separate networks. However, the comparison of these two tasks is limited by the fact that the emotional counting Stroop task does not involve true interference effect [6]. Indeed, in both tasks, the subjects report, through button press, the number of words (1–4) that appear on a screen. However, in the emotional counting task, interference trials contain emotional words (e.g., “murder” written three times), whereas in the non-emotional counting Stroop interference trials contain number words that are incongruent with the correct response (e.g., “two” written three times) [2], [5]. Thus, while emotional counting task primarily measures the ability of emotional words (distractors) to withdraw attention from the main task (counting of words), the non-emotional task measures selective attention and how easily a person can suppress a habitual response, such as reading, in favor of a less familiar task, such as counting.

The current study was designed primarily to examine how the networks related to emotional interference triggered by semantic incompatibility between emotional target and emotional distractor differ from those involved in non-emotional interference. We took into account the limitations (lack of stable behavioral interference effect) of previous studies applying emotional analogs of interference tasks [1]–[4] and suggested emotional and non-emotional interference tasks in which the interference effect was based on semantic incompatibility between target and distractor. In order to make the tasks comparable, we proposed two distractor-specific interference conflicts whereby the emotional task required focusing on emotional features of the face and the non-emotional task on the non-emotional features. Consequently, in both tasks, we used the same emotional faces as targets with two different assignments – emotional recognition and age judging. In the emotional word-face interference task used in our study, stable behavioral interference effect arises from semantic incompatibility between task-relevant (recognition of emotional face) and task-irrelevant (reading of emotional word) information, and in the non-emotional word-face interference task, the conflict effect ensues from semantic incompatibility between task-relevant (judgment of the person's age) and task-irrelevant information (reading of a word whose meaning does not fit the target's age category). Thus, both tests measure selective attention and how easily a person can suppress a habitual response, such as reading, in favor of a less familiar task, such as recognition of facial expression or age judging.

We sought to investigate the effect of emotional compared to non-emotional distractors on the processes of interference resolution and expected, based on results from previous studies, emotional distractors to produce increased interference effect. We applied the tasks to test for two possible outcomes pertaining to increased distractibility prompted by emotional words. First, on the basis of evidence suggesting dissociable effect of emotion on brain activity [7], whether the emotional distractor would increase activity in brain regions responsible for emotional processing (amygdala, ventrolateral and medial prefrontal cortices, rostral ACC) while simultaneously decreasing activity in the regions responsible for conflict resolution processes (dorsal ACC, dorsolateral prefrontal cortex). Second, if any interference task (whether triggered by an emotional or non-emotional distractor) based on the semantic incompatibility between target and distractor would also involve networks associated with interference resolution in general. We deemed it likely that any interference (whether triggered by an emotional or non-emotional distractor) would also increase demands for executive control to suppress the distracting information. This, in turn, would suggest the involvement of overlapping networks associated with interference, whether triggered by emotional or non-emotional distractors. Like the networks underlying this function in the non-emotional interference Stroop tasks, we expected to see the involvement of the dorsal ACC, the dorsolateral prefrontal cortex, the inferior frontal gyrus, the posterior parietal cortex, and the anterior insula [5], [8]. We also sought to determine if these networks are capable of dynamic adjustments based on the increased demands exerted by emotional distractors. Finally, the goal was to prove whether interference resolution in the emotional task would be facilitated by the target's emotional salience.

## Materials and Methods

The participants were students from Aachen University, recruited by means of an advertisement. 24 healthy right-handed participants (12 women and 12 men), all native speakers of German, took part in the study. Participants with neurological, psychiatric or other medical illnesses with impact on brain functioning were excluded. Previous head injuries with loss of consciousness and substance abuse or dependence were the additional exclusion criteria. Demographic variables and other criteria for comparison are summarized in table 1. All participants were screened by an experienced psychiatrist (NC) from the university hospital Aachen. The screening included a short version of the structured clinical interview for Axis I disorder (SCID-I, German version [9]) and an interview during which the participants' medical history was recorded. During the latter, participants with Axis II disorders were identified and excluded. The decision was based on clinical observation, information (obtained during the interview) related to the subjects' life events, typical behavior and relationships, their inner experience and capacity for self-reflection.

**Table 1:** Demographic and neuropsychological characterizations.

Group characteristics and demographic data	Mean values and standard deviations
Subject numberGender	2412 female, 12 male
Age (years)	27.6±4.1
Mean education (years)	17.0±1.4
Processing speed (TMT-A, s)	19.7±4.1
Cognitive flexibility (TMT-B, s)	36.5±8.0
Verbal intelligence (WST, IQ)	104.6±10.6
Facial recognition (BFRT – hits, %)	46.2±4.2
Emotion discrimination (PERT – hits, %)	30.5±5.08

A detailed description of the study protocol was provided and all participants gave written informed consent. The study protocol was approved by the Institutional Review Board of the Medical Faculty, RWTH Aachen University. All subjects received a standardized instruction regarding the paradigm and a 5-minute training session outside the scanner. The training session included 2.5-minute presentations of the first and the second tasks, whereas the stimuli from both tasks were presented randomly. The aim was to explain the tasks to the participants so that they would know how to respond by pressing buttons.

Functional imaging was performed on a 3T Trio MR scanner (Siemens Medical Systems, Erlangen, Germany) using echo-planar imaging sensitive to BOLD contrast (voxel size: 3.0×3.0×3.0 mm^3^, 64×64 matrix, FoV: 192 mm^2^, 34 slices, gap 0. 75 mm, TR 2 s, TE 28 ms, α = 77°). The scans were acquired in interleaved mode.

Between two runs, we took a short break in order to introduce the subsequent task once again, thus enabling participants to focus attention on the new requirements.

### Interference tasks

**Figure 1:**
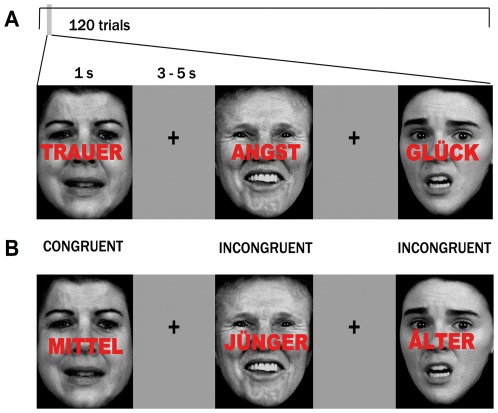
Non-emotional and emotional conflict paradigms. (A) Basic stimulus material consisting of congruent and incongruent face expression/word pairs from the FEBA faces collection for emotional paradigm. (B) Analogous to non-emotional conflict task, basic stimulus material for non-emotional paradigm.

In the emotional interference task, combinations of an emotional face in the background (with a sad, or fearful, or happy facial expression) with one of the following words (distractors) ‘TRAUER’, ‘ANGST’, and ‘GLÜCK’ (German for “sadness”, “fear” and “happiness”) printed across the face in bold red letters represented emotionally congruent or incongruent stimuli. The participants were asked to judge the emotion of the faces while trying to ignore the words. In the non-emotional interference task, the same emotional faces were presented in the same order with the words “JÜNGER”, “MITTEL” or “ÄLTER” (German for “younger”, “middle-aged” or “older”) superimposed in red letters, producing age-congruent and -incongruent stimuli. Subjects were required to classify the faces as being young (20 years or younger), middle-aged (middle 30 s to middle 40 s) or older (older than 60 years of age), while trying to ignore the task-irrelevant distractor. Except for the distractors, the paradigm remained constant (see figure 1).

120 trials were presented in one run. The trials were classified as congruent (C) or incongruent (I). The number of congruent and incongruent trials, the number of faces belonging to each emotional or age category, and the number of face-word combinations were counterbalanced for both tasks. Participants were instructed to identify the facial expression and answer as quickly and precisely as possible by pressing one of the three answer buttons with the right index, middle or ring finger for sad, fearful or happy faces, or for younger, middle-aged or older age categories respectively.

Images of the faces were taken from the set used in Facial Emotions for Brain Activation (FEBA) test [10] and put in standardized positions of the eyes and the mouth and normalized brightness. Faces were displayed for 1000 ms with randomized interstimulus interval (4.00±0.38 s (mean±SD), range 3–5 s) using Presentation software (Neurobehavioral Systems, San Francisco, USA). Between face presentations, a fixation cross was shown. Participants watched the pictures via video goggles (VisuaStim XGA, Resonance Technology Inc., Los Angeles, USA) and gave responses via LUMItouch response system (http://ucdirc.ucdavis.edu/techsupport/Lumitouch_brochure.pdf).

Reaction times (RT) were collected during the fMRI experiment. Error trials (wrong answers and omissions) were excluded from the analysis. For accuracy calculations, all types of errors were considered.

### Image preprocessing

Images were processed using Statistical Parametric Mapping (SPM) software (version SPM5, http://www.fil.ion.ucl.ac.uk/spm) on a Linux workstation. The first five images of each time series were excluded due to T1 stabilization effects. All remaining images were slice-time corrected and realigned to the first image. Images were normalized to a standard EPI template (interpolation to 2×2×2 mm^3^ resolution) and smoothed with an isotropic Gaussian kernel (8 mm full width at half maximum).

For each subject, two different first-level models for fMRI data were estimated in order to perform two analyses on the second level with different emphases. The first model sought to identify brain regions that are sensitive to the congruency effect of the presented face and the superimposed word, while, concurrently, modeling the three different emotions of the faces. These effects were estimated for each task (emotional and non-emotional) separately. Thus, the two sessions were modeled with 12 regressors of interest: 2 task (emotional vs. non-emotional) by 2 conflict (congruent vs. incongruent) by 3 facial expression (sad, fear, happy). Delta-functions with the time-points of presentation of the trials of each type were convolved with the canonical hemodynamic response function (HRF) to build regressors for the model of the time-series. The first-level model also included an additional (HRF-convolved) regressor of no interest for error trials (wrong answers and omissions) and an intercept for the mean across each session. A high-pass filter with a cut-off period of 128 s was applied and serial auto-correlations were accounted for by including a first-order auto-regressive covariance structure (AR(1)).

Contrast estimates of the 12 regressors of interest from each subject were entered in a three-way ANOVA with dependent observations (task (2 levels) by congruency (2 levels) by facial expression (3 levels)). The main goal of the current study was to show differences and commonalities between brain regions related to resolving conflict during two different tasks. To this end, we first calculated contrasts for the task by conflict interaction in order to stress the differences. Second, we present the conflict contrasts for each single task independently, for illustrative purposes. Finally, in order to assess the commonalities, we also present conjunctions of conflict contrasts which are described in greater detail below.

Unless otherwise stated, the significance level for all main effects of the imaging data was set to p<0.05 corrected at the cluster-level using a cluster-defining threshold of p<0.0001 at the voxel-level. This is also true for the correlational analyses, which were performed in order to reveal brain regions associated with reaction times. The contrast for the task by conflict interaction was likewise thresholded at p<0.05 corrected at the cluster-level. Due to the reduced sensitivity of interaction as opposed to the main effects, however, we decided to perform the correction of the task by conflict interaction on clusters formed at p<0.001 uncorrected.

Apart from showing differences between the conflict contrasts we also stress the commonalities of different (independent) contrasts of incongruent vs. congruent items. For this purpose we calculated the conflict contrasts for each level of the different faces and the two tasks, i.e. six of these conflict contrasts were created. In order to assess what all of these contrasts had in common, we put these six contrasts in conjunction thresholded at p<0.05 uncorrected [11]. This liberal threshold accounts for the fact that a voxel to be declared significant had to pass six simultaneous tests at this threshold, with the probability of six independent events occurring simultaneously when the probability of each single event is 0.05 equals 0.05^6^ or 1.5625e-8. In addition, we show the three conflict contrasts from the emotional task in conjunction at the threshold of p<0.05 uncorrected. Again, the probability of three independent events occurring simultaneously when the probability of each single event is 0.05 equals 0.05^3^ or 0.000125. It is stressed that the nominal significance level is p<0.05 uncorrected for each of the conjunctions and that the calculation of the joint probabilities is just for illustrative purposes. By showing these conjunctions, we aimed at identifying regions that are involved in each single conflict contrast simultaneously.

Correlation analyses determined the relationship between activation strength in response to incongruent (as compared to congruent) trials in single-subject contrasts on one hand and individual conflict-related reaction time slowing (difference in reaction time in response to incongruent as compared to congruent trials) on the other. This analysis was done separately for emotional and non-emotional tasks.

The resulting SPM(T) and correlation maps were anatomically localized using version 1.5 of the SPM Anatomy toolbox (http://www.fz-juelich.de/ime/spm_anatomy_toolbox, [12], [13]).

## Results

### Behavioral results

For a three-way *task* × *congruency* × *face* RTs analysis of variance (ANOVA), items were assigned to each level of the factors *task* (two levels) × *congruency* (two levels) and *face* (three levels) The analysis revealed the *task* × *congruency* interaction (*F*_1,23_ = 20.0, p<0.001) since the interference effect was stronger in the task based on word-emotion than in the one based on word-age interference (164 ms vs. 82 ms). There was also significant *task* × *face* interaction (*F*_2,46_ = 16.5, p = 0.001).

In order to assess the putative effects of emotion or age categories on RTs, we also performed two-way ANOVA separately for each of the two tasks, first with the factors *emotion* (three levels: sadness, fear, happiness) and *congruency* (two levels) and then with the factors *age* (three levels: younger, middle-aged, older) and *congruency*. In the emotional task, an *emotion* × *congruency* analysis of variance on RT data revealed a significant effect of the factor *congruency* (*F*_1,23_ = 58.4, p<0.001), as incongruent stimuli were processed more slowly (for descriptive statistics see table 2). The same analysis also revealed a significant effect of the factor *emotion (F*_2,46_ = 22.2, p<0.001). As post hoc analysis revealed, incongruent stimuli with happy facial expressions were associated with faster RTs as compared to the incongruent stimuli with sad (or fearful expressions (t_23_ = −8.5, p<0.001 and t_23_ = −6,1, p<0.001 respectively). The same applied to the congruent stimuli (t_23_ = −5.5, p<0.001 and t_23_ = −4,0, p = 0.001 respectively) (table 2). In the non-emotional interference task, only a significant main effect of congruency was detected (*F*_1,23_ = 20.7, p<0.001: 967 ms±165 ms vs. 886 ms±163 ms (mean±SD) for incongruent and congruent stimuli respectively).

**Table 2:** Descriptive statistics of behavioral data in emotional task.

Congruency	Emotion	RT (ms)	SD
CONGRUENT TRIAL	FEARFUL	989	211
	HAPPY	791	160
	SAD	963	145
INCONGRUENT TRIAL	FEARFUL	1123	294
	HAPPY	959	213
	SAD	1152	242

A three-*way task* × *congruency* × face accuracy analysis of variance (ANOVA) revealed a significant main effect of task (*F*_1,23_ = 114,5, p<0.001) as the emotional task was associated with a higher level of correct responses (93% vs. 78%). In order to assess the putative effects of emotion or age categories on accuracy, we also performed two-way ANOVA separately for each of the two tasks, first with the factors *emotion* (three levels: sadness, fear, happiness) and *congruency* (two levels) and then with the factors *age* (three levels: younger, middle-aged, older) and *congruency*. In the emotional task, an *emotion* × *congruency* accuracy analysis of variance on RT data revealed a significant effect of the factors *congruency (F*_1,23_ = 11.3, p = 0.004; 95% vs. 91% for congruent and incongruent stimuli respectively) and *emotion (F*_2,46_ = 3.9, p = 0.040) as targets with happy expressions were associated with a higher level of accuracy (96% of all happy faces, 91% of all sad faces and 92% of all fearful faces were correctly recognized). In the non-emotional task, there was significant effect only of the factor *congruency (F*_1,23_ = 15.0, p = 0.001; 80% vs.75% for congruent and incongruent stimuli respectively.

### FMRI results

#### Differences in response to conflict between tasks

**Figure 2:**
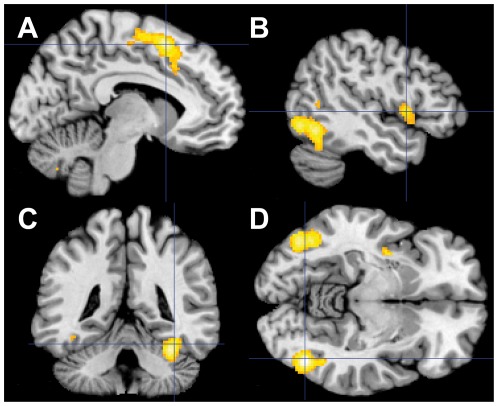
Areas recruited more strongly in response to emotional as compared to non-emotional conflict. (A) The emotional interference task, as compared to the non-emotional task, led to a stronger recruitment of the posterodorsal medial frontal cortex encompassing Brodman areas 8, 6, 32 and 24. The intersection is in left SMA (MNI coordinates: x = −8, y = 12, z = 54). (B) Stronger recruitment of left IFC/anterior insula region (MNI coordinates: x = −48, y = 8, z = 0) and the left occipitotemporal visual cortex. (C) Recruitment of the right fusiform face area (MNI coordinates: x = 36, y = −44, z = −22). (D) Bilateral recruitment of the bilateral occipitotemporal visual cortex. Intersection is in the right inferior temporal gyrus (MNI coordinates: x = 44, y = −68, z = −10). Activity is shown at P<0.05 (cluster-level family-wise error-corrected; cluster-forming threshold at voxel-level P<0.001).

The emotional compared with the non-emotional interference task led to a stronger recruitment of the posterodorsal medial frontal cortex (pMFC) in the region where Brodmann areas 8, 6, 32 and 24 border one another. Another cluster of significant activation included the left inferior frontal cortex (IFC) with the adjunct left anterior insula. The bilateral occipitotemporal visual cortex, including the bilateral fusiform gyri, the bilateral inferior and middle temporal gyri, and the bilateral inferior and middle occipital gyri, was also recruited more strongly. The same applied to the left superior (SPL) and the left inferior parietal lobules (IPL), and the bilateral cerebellum (Figure 2 and Table 3).

**Table 3:** Brain regions that are more strongly involved in response to conflict (I>C) in emotional compared to non-emotional task at P<0.05 cluster-level family-wise error-corrected; cluster-forming threshold at voxel-level P<0.001.

Anatomical region	Side	k	FWE-corrected P_cluster_	Peak voxel			
				T	x	y	z
SMA (BA 6)	L	1946	P<0.001	5.54	−8	12	54
SMA (BA 6)	R			4.56	4	14	50
(cluster extends to bilateral Anterior Cingulate and bilateral BA 8)							
Inferior Occipital Gyrus (BA 19)	L	1289	P<0.001	5.32	−44	−72	−10
Cerebellum (VI)	L			5.26	−32	−56	−28
Fusiform Gyrus (BA 37)	L			4.30	−42	52	−18
(cluster extends to Inferior Temporal Gyrus and Middle Occipital Gyrus)							
Cerebellum (VIII)	R	565	P<0.001	5.51	8	−70	−40
Cerebellum (VI)	R	455	P<0.001	5.91	34	−48	26
Fusiform Gyrus (BA 37)	R			4.92	36	−44	−22
Insula (BA 13)	L	412	P<0.001	4.40	−38	4	−2
Inferior Frontal Gyrus (BA 44)				4.27	−48	8	0
Inferior Temporal Gyrus (BA 20)	R	376	P<0.001	6.14	44	−68	−10
Inferior Occipital Gyrus (BA 19)	R			5.04	44	−72	−10
(cluster extends to right Middle Occipital Gyrus)							
Superior Parietal Lobule (BA 7)	L	258	P<0.001	5.13	−22	−72	60
Precentral Gyrus (BA 6)	L	211	P<0.001	4.29	−42	−6	62
Inferior Frontal Gyrus (BA 45)	L	190	P = 0.001	4.52	−48	30	16
Inferior Parietal Lobule (BA 40)	L	160	P = 0.002	4.10	−32	−50	46

The opposite contrast showed differences between the tasks in the right superior and middle frontal gyri (Table S1). This effect was seen at whole brain P<0.001 uncorrected.

#### Effect of both types of conflict on BOLD response

As the conjunction analysis reveals, the anterior dorsal anterior cingulate (dACC), the bilateral anterior insula with the adjacent inferior frontal cortex (IFC), the bilateral supplementary motor areas (SMA) and the left thalamus were recruited more strongly in response to incongruent compared to congruent trials in both tasks. Results are presented in Table 4.

**Table 4:** Brain regions involved in each single conflict contrast (I>C), independent of the task and the facial expression.

Anatomical region	Side	k	Peak voxel			
			T	x	y	z
Anterior Cingulate (BA 32)	L	184	2.47	−4	26	34
Anterior Cingulate (BA 32)	R		2.34	6	24	30
Inferior Frontal Gyrus (BA 44)	L	68	2.54	−44	−8	6
Insula	L		2.27	−43	6	5
SMA (BA 6)	R	15	2.01	8	16	66
SMA (BA 6)	L	14	1.88	−2	12	54
Insula	R	11	1.98	34	32	0
Insula	R	11	2.04	34	20	−12
Inferior Frontal Gyrus (BA 44)	R		1.82	34	28	−12
Thalamus	L	10	2.03	−18	−18	10

The results are based on a conjunction across six contrasts thresholded at p<0.05 uncorrected (see text for justification of the threshold).

#### Effect of emotional conflict on BOLD response

**Figure 3:**
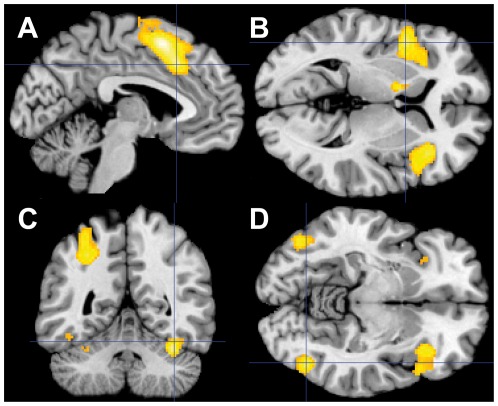
Effect of emotional conflict. (A) Involvement of left SMA and the left dACC. The intersection is in the left dACC (MNI coordinates: x = −4, y = 20, z = 38). (B) Response of the bilateral anterior insula/IFC region. The intersection is in the left anterior insula (MNI coordinates: x = −46, y = 8, z = 2). (C) Recruitment of the right fusiform face area (MNI coordinates: x = 36, y = −52, z = −22) and the left inferior parietal lobule. (D) Bilateral recruitment of the bilateral occipitotemporal visual cortex. Intersection is in the right temporal gyrus (MNI coordinates: x = 46, y = −68, z = −10). Activity is shown at p<0.05 FWE corrected with a cluster size >20 voxels.

At a whole-brain FWE correction of P<0.05 with a cluster extent of 20 voxels, in response to incongruent compared to congruent trials, significant BOLD signal increased in the dACC, the bilateral SMA and the left precentral gyrus. Within the bilateral occipitotemporal visual cortex, activation was seen in the bilateral fusiform gyrus, the bilateral inferior temporal and the bilateral inferior occipital gyri. The bilateral anterior insula/bilateral IFC region was also more strongly recruited. The same applied to the left pallidum and the bilateral cerebellum. Results are presented in Figure 3 and Table 5. As the conjunction analysis demonstrated, those regions responded to emotional conflict independent of the type of emotional target (Table S2). In addition, the conjunction analysis revealed involvement of the right middle temporal and bilateral middle occipital gyri, the bilateral thalamus and the right caudate nucleus in response to emotional conflict.

**Table 5:** Brain regions involved during emotional task in response to interference conflict at P<0.05 voxel-level family-wise error-corrected.

Anatomical region	Side	k	Peak voxel			
Positive BOLD response			T	x	y	z
SMA (BA 6)	L	2014	9.41	0	12	52
Anterior Cingulate (BA 32)	L		8.03	−4	20	38
Anterior Cingulate (BA 32)	R		7.01	10	18	38
SMA (BA 6)	R		5.83	8	14	66
Precentral Gyrus (BA 6)	L	984	7.73	−54	2	40
Inferior Frontal Gyrus (BA 44)	L		6.76	−42	10	26
Inferior Frontal Gyrus (BA 45)	L		6.50	−48	30	16
Inferior Parietal Lobule	L	937	7.14	−32	−48	44
Superior Parietal Lobule (BA 7)	L		6.54	−26	−64	48
Postcentral Gyrus (BA 1)	L		5.92	−46	−36	60
Insula (BA 13)	R	861	7.84	34	26	−4
Temporal Pole	R		7.55	44	16	−2
Inferior Frontal Gyrus (BA 45)	R		5.94	52	18	−12
Insula (BA 13)	L	774	7.69	−46	8	2
Precentral Gyrus (BA 6)	L	396	6.25	−36	−4	64
Cerebellum (VI)	R	328	7.97	34	−52	−26
Fusiform Gyrus (BA 37)	R		6.70	36	−52	−22
Inferior Occipital Gyrus (BA 19)	L	253	6.44	−46	−72	−8
Fusiform Gyrus (BA 37)	L		5.89	−46	−60	−16
Inferior Temporal Gyrus (BA 20)	L		5.27	−46	−50	−16
Inferior Temporal Gyrus (BA 20)	R	156	7.57	46	−68	−10
Inferior Occipital Gyrus (BA 19)	R		6.04	46	−72	−8
Pallidum	L	56	6.26	−12	0	2
Cerebellum (VIII)	R	28	5.73	8	−70	−40
Cerebellum (VI)	L	20	5.28	−30	−54	−28

Negative BOLD response			T	x	y	z
Angular gyrus	R	84	5.84	50	−70	40

In response to congruent as compared to incongruent trials, there was stronger involvement of the right angular and right superior frontal gyri (Table 5). This effect was seen at whole brain FWE correction of P<0.05 with a cluster extent of 20 voxels.

#### Effect of non-emotional conflict on BOLD response

**Figure 4:**
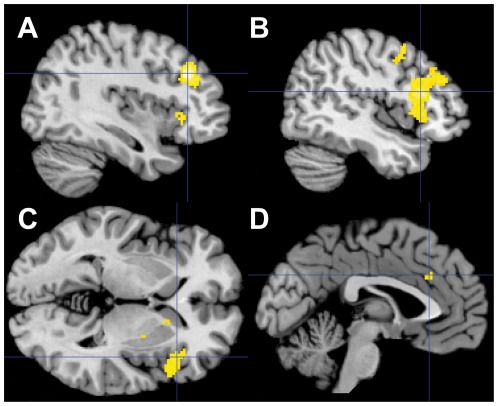
Effect of non-emotional conflict. (A) Recruitment of the right middle frontal gyrus (MNI coordinates x = 38, y = 30, z = 30). (B) Recruitment of the right inferior frontal gyrus (MNI coordinates x = 46, y = 20, z = 16). (C) Recruitment of the right anterior insula (MNI coordinates x = 42, y = 22, z = −2). D) Recruitment of dACC (MNI coordinates x = −2, y = 28, z = 32). Activity is shown at P<0.05 (cluster-level family-wise error-corrected; cluster-forming threshold at voxel-level P<0.0001).

At a whole-brain FWE correction of P<0.05 without a cluster extent, in response to incongruent compared to congruent trials, significant BOLD signal increased in the right inferior frontal gyrus (MNI x = 46 y = 20 z = 16; 57 voxels) and the right middle frontal gyrus (MNI x = 38 y = 30 z = 30; 56 voxels). For exploratory analysis, we also performed analysis at P<0.05 corrected at cluster level, voxel-wise inclusion threshold at p<0.0001, which revealed responses to non-emotional conflict in the right putamen, the right pallidum and the right anterior insula. Further BOLD signal was observed in the right dACC (BA 32). Results are presented in Figure 4 and Table 6.

**Table 6:** Activated brain regions during non-emotional task in response to interference conflict at P<0.05 cluster-level family-wise error-corrected; cluster-forming threshold at voxel-level P<0.0001.

Anatomical region	Side	k	FWE-corrected	Peak voxel			
				T	x	y	z
Middle Frontal Gyrus	R	1048	P<0.001	6.31	38	30	30
Inferior Frontal Gyrus (BA 44)	R			5.49	46	20	16
Inferior Frontal Gyrus (BA 45)	R			5.17	50	24	10
Insula	R			4.77	42	22	−2
Putamen	R	80	P = 0.010	4.71	16	12	−6
Putamen	R	68	P = 0.017	4.90	28	−4	6
Pallidum	R			4.89	26	−2	2
Precentral Gyrus	R	52	P = 0.039	4.27	44	2	42
Anterior Cingulate	L	41	P = 0.052	4.20	−2	26	30

The opposite contrast (congruent trials > incongruent trials) did not reveal any significant differences.

#### Correlation between behavioral interference and neuroimaging data

In the emotional task, the stronger interference effect was associated with a network that included the left amygdala, the right thalamus, the right insula, the bilateral lateral prefrontal cortex (LPFC), the bilateral SMA, the right anterior temporal cortex, the bilateral posterior temporal-occipital cortex, and the bilateral supramarginal gyrus (Table S3). In the non-emotional task, the stronger the behavioral interference effect, the more vigorous was the recruitment of the bilateral IFC and the left IPL (Table S4).

No negative correlations were detected in either task.

Finally, we tested if there were differences in response to conflict between the genders (a congruency-by-gender interaction) for each of the tasks separately. Comparing, first, the emotional and, then, the non-emotional columns of the design between the two groups (i.e. two directional t-contrasts incongruent vs. congruent during emotional task in the female group larger than incongruent vs. congruent during emotional task in the male one, and vice versa) we did not find any differences between the genders at p<0.001 uncorrected.

## Discussion

We applied emotional and non-emotional variations of the Stroop task using face stimuli as targets and word stimuli as distractors. In both versions of the task, emotional and non-emotional distractors generated significant interference conflicts. In the emotional task, the interference effect was greater than in the non-emotional one, owing to high levels of distraction caused by emotional words. There were, however, fewer errors overall in the emotional compared to the non-emotional task, suggesting that when it came to targets, attention was likely facilitated by the emotional features of the faces based on which participants were required to make their judgment. Thus, cognitive functions were influenced by the emotional significance of stimuli, causing increased accuracy as a benefit and increased distractibility as a detrimental effect on the ongoing behavior. A parallel increase of conflict-related activity was seen in the functional systems associated with conflict monitoring and attention. While both tasks engaged the primary visual cortex, it was conflict in the emotional task (entailing focus on emotions) that led to the involvement of the ventral cortical region including the bilateral fusiform gyri (with the fusiform face area) and the bilateral inferior and middle occipital gyri. Thus, interference conflict engaged areas associated with visual attention and high-level visual processing in order to augment the evaluation of emotion-laden information. Task-sensitive activity in the occipitotemporal visual cortex in response to emotional conflict is in line with the notion that attention is guided by an object's affective significance [14], [15]. The involvement of the fusiform face area suggests that the resolution of conflict was likely facilitated by an enhanced processing of emotionally laden information causing more pronounced emphasis on target. Thus, despite increased distractibility caused by emotional words, goal-specific emotionally salient information helped maintain focus on target. As a consequence, accuracy in the emotional task was higher than in the non-emotional task. These findings also corroborate the idea that the affective processing of a stimulus is linked to cognitive factors such as attention and does not occur automatically [16]. Thus, despite using the same emotional faces, only the task-relevant attended emotional stimuli led to the involvement of the ventral extrastriate cortex. On the other hand, an elevated need for adjustment in cognitive control to overcome the conflict triggered by emotional words was seen in a stronger involvement of the functionally integrated unit associated with the monitoring-performance mechanism and detection of response conflict (cingulate and paracingulate, association and premotor cortices) [17]. Further areas of significantly stronger activation were identified in the left inferior frontal gyrus abutting the anterior insula (emotional processing [18], [19], implementation of inhibitory processes [20] and in the temporoparietal junction (target detection [21]). We also observed robust involvement of the cerebellum, a region typically thought of as a motor center. Similarly, previous research has suggested the involvement of the cerebellum in mediating conflict resolution processes with participants showing impaired capacity for conflict resolution following cerebellar lesions (despite an intact prefrontal cortex) [22]. Thus, successful resolution of emotional conflict demonstrates both acceleration and dynamic adjustments of systems in response to elevated conflict-associated demands. It can therefore be suggested that in spite of higher levels of distraction produced by emotional words, the networks involved in conflict resolution succeeded in effective allocation of attentional and control resources to achieve efficient goal-directed behavior.

The adjustment of conflict resolution processes was also seen on the individual level. Thus, in the emotional task, individual conflict-related reaction time slowing correlated positively with a complex network including the left amygdala, the right thalamus, the bilateral insula/LPFC region, the right anterior temporal cortex, the bilateral posterior temporal-occipital cortex, and the bilateral supramarginal gyri. This network indicates a relationship of higher recruitment of attentional, inhibitory and conflict processing recourses as a response to higher levels of distractibility triggered by emotional words. The link between the difficulty to ignore an emotional distractor and amygdala activity might also endorse the view that the amygdala allocates attentional resources to stimuli required for prioritizing particular features of information processing [15].

Contrary to findings suggesting specific involvement of the IFC in the inhibition of distracting emotions [18], in both tasks participants with stronger interference effects also revealed stronger activation in the IFC. These results are in line with studies that suggest that the IFC's primary role is to select among multiple representations the best to serve the task at hand [23] and further support the notion of the region's general involvement in the inhibitory function.

In both tasks, the interference conflict initiated strongly overlapping networks including the regions involved in non-emotional interference [5], [8]. Thus, we saw conjoint recruitment of processing modules including sensory turning, selective attention during response selection, inhibition (bilateral IFC and insula), premotor planning (bilateral SMA), vigilance, error response monitoring and resolution of response conflict (dACC), and, finally, a motor output (thalamus, motor portion of cingulate cortex) in response to interference conflict in both tasks. Based on this observation, the presence of a general conflict network irrespective of the character of stimulus or distractor can be suggested. For emotional and non-emotional monitored events, the most pronounced cluster of activations is in Brodmann Area 32, which further underscores the role of this area in the unified performance monitoring function [5], [17], [24], [25].

Taken together, our results suggest that the resolution of interference triggered by emotional and non-emotional distractors involves the same systems, which are capable of flexible adjustments based on conflict demands. Contrary to the notion that increased interference prompted by emotional words results in diminished cognitive control, our findings indicate dynamic adjustments of the control processes in response to increased distractibility. The evaluation of a task-relevant object's significance in the occipitotemporal cortex appears to play a pivotal role in signaling the necessity for such adjustments of executive functions. Thus, the attentional system being capable of evaluating a target's significance, it helps maintain focus, despite increased distraction, when the target is deemed to be of particular relevance. Moreover, the involvement of the extrastriate cortex in response to emotional conflict suggests that it is not only inhibition of the distractor but also target amplification that is a facilitative mechanism of conflict resolution. Also, target amplification seems to take place when the target (f.e. emotional content) is of particular significance. This issue, however, was beyond the scope of the present study, which was focused on interference conflict, and ought to be explored in future research. Finally, contrary to the notion of a functional segregation of brain areas into ventral (emotional) and dorsal (cognitive) systems, our findings tend to endorse the view of a cognitive-emotional integration [15] in the executive control functions. As both tasks involved a broad network of frontal and prefrontal regions in response to conflict, the tasks can be applied to study the adjustment capabilities of those systems stressed by different conflict demands. These observations are likely to be of great interest in the context of psychiatric disorders.

As a limitation of the study, however, we must cite the relatively low number of trials per condition (congruent or incongruent face-word combinations) used in the study −20 trials, as opposed to the previously recommended 25, although this critically depends on the expected effect size and therefore also on task and brain region [26]. However, as we did not detect any significant effect of the target type (e.g. emotional face or age category) on the interference effect, we compared congruent and incongruent trials independently of their emotional (sad, fearful or happy) or non-emotional (young, middle-aged or old) category, putting the trials in simply “emotional” or “non-emotional” category. It means that in each task the number of trials per condition (congruent vs. incongruent) was 60. In any case, given that even false responses can affect the number of trials, it should be noted that statistical power might have been compromised in the present study. Enhanced power and thus avoidance of false negative results can be expected in future replication studies if more trials per condition are added.

A further limitation of the study is the lack of a structural (T1) scan. It should be noted, therefore, that the absence of anatomical coregistration is likely to have a bearing on the information vis-à-vis the localization of specific brain structures.

## Supporting Information

Table S1 **Brain regions that are more strongly involved in response to conflict (I>C) in non-emotional compared to non-emotional task at P<0.001 uncorrected.**
(DOC)

Table S2 **Brain regions involved in each single conflict contrast (I>C) in the emotional task, independent of the facial expression.** The results are based on a conjunction across three contrasts thresholded at p<0.05 uncorrected (see text for justification of the threshold).
(DOC)

Table S3 **Areas that positively correlated with conflict-related slowing during incongruent trials in the emotional task at P<0.05 cluster-level family-wise error-corrected; cluster-forming threshold at voxel-level P<0.0001.**
(DOC)

Table S4 **Areas that positively correlated with conflict-related slowing during incongruent trials in the non-emotional task at P<0.05 cluster-level family-wise error-corrected; cluster-forming threshold at voxel-level P<0.0001.**
(DOC)

## References

[1] Compton RJ, Banich MT, Mohanty A, Milham MP, Herrington J (2003). Paying attention to emotion: an fMRI investigation of cognitive and emotional stroop tasks.. Cogn Affect Behav Neurosci.

[2] Whalen PJ, Bush G, McNally RJ, Wilhelm S, McInerney SC (1998). The emotional counting Stroop paradigm: a functional magnetic resonance imaging probe of the anterior cingulate affective division.. Biol Psychiatry.

[3] Whalen PJ, Bush G, Shin LM, Rauch SL (2006). The emotional counting Stroop: a task for assessing emotional interference during brain imaging.. Nat Protoc.

[4] Williams JMG, Mathews A, MacLeod C (1996). The emotional Stroop task and psychopathology.. Psychol Bull.

[5] Bush G, Whalen PJ, Rosen BR, Jenike MA, McInerney SC (1998). The counting Stroop: an interference task specialized for functional neuroimaging: Validation study with functional MRI.. Hum Brain Mapp.

[6] Etkin A, Egner T, Kalisch R (2011). Emotional processing in anterior cingulate and medial prefrontal cortex.. Trends Cogn Sci.

[7] Mayberg HS (1997). Limbic-cortical dysregulation: a proposed model of depression.. J Neuropsychiatry Clin Neurosci.

[8] Peterson BS, Skudlarski P, Gatenby JC, Zhang H, Anderson AW (1999). An fMRI study of Stroop word-color interference: evidence for cingulate subregions subserving multiple distributed attentional systems.. Biol Psychiatry.

[9] Wittchen H.-U (1997). SKID-I..

[10] Gur RC, Sara R, Hagendoorn M, Marom O, Hughett P (2002). A method for obtaining 3-dimensional facial expressions and its standardization for use in neurocognitive studies.. J Neurosci Methods.

[11] Nichols T, Brett M, Andersson J, Wager T, Poline JB (2005). Valid conjunction inference with the minimum statistic.. Neuroimage.

[12] Eickhoff SB, Stephan KE, Mohlberg H, Grefkes C, Fink GR (2005). A new SPM toolbox for combining probabilistic cytoarchitectonic maps and functional imaging data.. Neuroimage.

[13] Eickhoff SB, Paus T, Caspers S, Grosbras MH, Evans AC (2007). Assignment of functional activations to probabilistic cytoarchitectonic areas revisited.. Neuroimage.

[14] Phelps EA, Ling S, Carrasco M (2006). Emotion facilitates perception and potentiates the perceptual benefits of attention.. Psychol Sci.

[15] Pessoa L, Adolphs R (2010). Emotion processing and the amygdala: from a ‘low road’ to ‘many roads’ of evaluating biological significance.. Nat Rev Neurosci.

[16] Pessoa L, Kastner S, Ungerleider LG (2002). Attentional control of the processing of neural and emotional stimuli.. Brain Res Cogn Brain Res.

[17] Ridderinkhof KR, Ullsperger M, Crone EA, Nieuwenhuis S (2004). The role of the medial frontal cortex in cognitive control.. Science.

[18] Dolcos F, McCarthy G (2006). Brain systems mediating cognitive interference by emotional distraction.. J Neurosci.

[19] Davis M, Whalen PJ (2001). The amygdala: vigilance and emotion.. Mol Psychiatry.

[20] Aron AR, Robbins TW, Poldrack RA (2004). Inhibition and the right inferior frontal cortex.. Trends Cogn Sci.

[21] Corbetta M, Kincade JM, Ollinger JM, McAvoy MP, Shulman GL (2000). Voluntary orienting is dissociated from target detection in human posterior parietal cortex.. Nat Neurosci.

[22] Schweizer TA, Oriet C, Meiran N, Alexander MP, Cusimano M (2007). The cerebellum mediates conflict resolution.. J Cogn Neurosci.

[23] Thompson-Schill SL, Jonides J, Marshuetz C, Smith EE, D'Esposito M (2002). Effects of frontal lobe damage on interference effects in working memory.. Cogn Affect Behav Neurosci.

[24] Botvinick MM, Cohen JD, Carter CS (2004). Conflict monitoring and anterior cingulate cortex: an update.. Trends Cogn Sci.

[25] Haas BW, Omura K, Constable RT, Canli T (2006). Interference produced by emotional conflict associated with anterior cingulated activation.. Cogn Affect Behav Neurosci.

[26] Murphy K, Garavan H (2005). Deriving the optimal number of events for an event-related fMRI study based on the spatial extent of activation.. Neuroimage.

