# Supplementary material for: Brain Circuitries Involved in Semantic Interference by Demands of Emotional and Non-Emotional Distractors
Source: PLoS One. 2012 May 29;7(5):e38155. doi: 10.1371/journal.pone.0038155 (PMC3362560; doi:10.1371/journal.pone.0038155)
Supplement: Table S1 — Brain regions that are more strongly involved in response to conflict (I>C) in non-emotional compared to non-emotional task at P<0.001 uncorrected. (DOC) [file pone.0038155.s001.doc]

**Table S1**

**Brain regions that are more strongly involved in response to conflict (I>C) in non-emotional compared to non-emotional task at P<0.001 uncorrected**

| **Anatomical region** | **Side** | **k** | **Peak voxel** | | | |
| --- | --- | --- | --- | --- | --- | --- |
|  |  |  | **T** | **x** | **y** | **z** |
| Angular gyrus | R | 98 | 4.10 | 50 | -70 | 40 |
| Superior medial gyrus | R | 48 | 4.19 | 10 | 44 | 48 |
| Middle frontal gyrus | R | 48 | 4.03 | 34 | 30 | 30 |
