# Supplementary material for: Brain Circuitries Involved in Semantic Interference by Demands of Emotional and Non-Emotional Distractors
Source: PLoS One. 2012 May 29;7(5):e38155. doi: 10.1371/journal.pone.0038155 (PMC3362560; doi:10.1371/journal.pone.0038155)
Supplement: Table S2 — Brain regions involved in each single conflict contrast (I>C) in the emotional task, independent of the facial expression. The results are based on a conjunction across three contrasts thresholded at p<0.05 uncorrected (see text for justification of the threshold). (DOC) [file pone.0038155.s002.doc]

**Table S2**

**Brain regions involved in each single conflict contrast (I>C) in the emotional task, independent of the facial expression. The results are based on a conjunction across three contrasts thresholded at p<0.05 uncorrected (see text for justification of the threshold)**

| **Anatomical region** | **Side** | **k** | **Peak voxel** | | | |
| --- | --- | --- | --- | --- | --- | --- |
| **Positive BOLD response** |  |  | **T** | **x** | **y** | **z** |
| SMA (BA 6)  Anterior Cingulate (BA 32)  Anterior Cingulate (BA 32) | L  L  R | 1452 | 3.32  3.32  2.70 | -8  -8  12 | 10  18  16 | 56  34  38 |
| Inferior Occipital Gyrus (BA 19)  Fusiform Gyrus (BA 37)  Inferior Temporal Gyrus (BA 20)  Middle Occipital Gyrus (BA 18) | L  L  L  L | 720 | 3.22  2.72  2.49  2.28 | -44  -46  -46  -38 | -72  -60  -48  -62 | -8  -16  -18  0 |
| Cerebellum (VI)  Fusiform Gyrus (BA 37) | R  R | 421 | 3.57  2.69 | 34  34 | -52  -48 | -15  -15 |
| Precentral Gyrus (BA 6)  Inferior Frontal Gyrus (BA 44) | L  L | 406 | 3.40  3.25 | -54  -60 | 0  8 | 38  32 |
| Insula (BA 13)  Inferior Frontal Gyrus (BA 47) | L  L | 317 | 3.30  2.23 | -44  -40 | 6  24 | 4  -8 |
| Precentral Gyrus (BA 6) | R | 247 | 2.50 | 38 | -48 | 52 |
| Inferior Temporal Gyrus (BA 20)  Inferior Occipital Gyrus (BA 19)  Middle Occipital Gyrus (BA 18) | R  R  R | 161 | 2.91  2.10  1.74 | 46  42  38 | -66  -78  -84 | -8  -6  2 |
| Insula (BA 13)  Inferior Frontal Gyrus (BA 45) | R  R | 99 | 2.62  2.27 | 38  36 | 18  32 | -14  -8 |
| Pallidum | L | 41 | 2.52 | -16 | 0 | 0 |
| Postcentral Gyrus (BA 1) | L | 27 | 2.07 | -46 | -34 | 64 |
| Thalamus | R | 25 | 2.00 | 12 | -12 | 0 |
| Middle Frontal Gyrus | L | 24 | 2.29 | -30 | 34 | 24 |
| Inferior Frontal Gyrus (BA 47) | R | 22 | 2.34 | 56 | 32 | 10 |
| Superior Frontal Gyrus | L | 21 | 2.26 | -12 | -8 | 80 |
| Insula (BA 13) | L | 21 | 2.51 | -36 | -4 | -12 |
| Middle Temporal Gyrus | R | 20 | 2.16 | 50 | -40 | 6 |
| **Negative BOLD response** |  |  | **T** | **x** | **y** | **z** |
| Angular Gyrus (BA 39) | R | 55 | 2.49 | 48 | -72 | 42 |
