# Supplementary material for: Brain Circuitries Involved in Semantic Interference by Demands of Emotional and Non-Emotional Distractors
Source: PLoS One. 2012 May 29;7(5):e38155. doi: 10.1371/journal.pone.0038155 (PMC3362560; doi:10.1371/journal.pone.0038155)
Supplement: Table S3 — Areas that positively correlated with conflict-related slowing during incongruent trials in the emotional task at P<0.05 cluster-level family-wise error-corrected; cluster-forming threshold at voxel-level P<0.0001. (DOC) [file pone.0038155.s003.doc]

**Table S3**

**Areas that positively correlated with conflict-related slowing during incongruent trials in the emotional task at P<0.05 cluster-level family-wise error-corrected; cluster-forming threshold at voxel-level P<0.0001**

| **Anatomical region** | **Side** | **k** | **FWE-corrected** | **Peak voxel** | | | |
| --- | --- | --- | --- | --- | --- | --- | --- |
|  |  |  |  | **T** | **x** | **y** | **z** |
| Inferior Frontal Gyrus (BA 44)  Inferior Frontal Gyrus (BA 45) | R  R | 443 | P<0.001 | 7.16  7.13 | 48  54 | 12  22 | 18  20 |
| Temporal Pole (BA 38)  Inferior Frontal Gyrus (BA 47)  Insula (BA 13) | R  R  R | 309 | P<0.001 | 7.00  6.96  5.83 | 46  42  34 | 20  18  24 | -18  -14  -2 |
| Inferior Frontal Gyrus (BA 45)  Middle Frontal Gyrus (BA 46) | L  L | 259 | P<0.001 | 6.61  5.80 | -48  -36 | 40  40 | 14  12 |
| Precentral Gyrus (BA 4)  Precentral Gyrus (BA 6) | R  R | 248 | P<0.001 | 7.47  5.54 | 38  40 | -14  -4 | 50  48 |
| Precentral Gyrus (BA 6)  SMA (BA 6) | L  L | 191 | P<0.001 | 5.71  5.66 | -24  -12 | -18  -8 | 72  74 |
| Supramarginal Gyrus (BA 40) | R | 187 | P<0.001 | 6.28 | 48 | -38 | 32 |
| Supramarginal Gyrus (BA 40)  Superior Temporal Gyrus (BA 22) | L  L | 186 | P<0.001 | 6.52  5.12 | -60  -56 | -38  -34 | 24  14 |
| Postcentral Gyrus (BA 6)  Precentral Gyrus (BA 6) | L  L | 164 | P<0.001 | 6.11  5.48 | -62  -60 | 2  2 | 20  32 |
| Inferior Frontal Gyrus (BA 47)  Middle Frontal Gyrus (BA 46) | R  R | 157 | P<0.001 | 7.13  5.38 | 46  44 | 36  52 | -8  6 |
| Calcarine Gyrus (BA 18)  Linual Gyrus (BA 17)  Fusiform Gyrus | R  R  R | 147 | P<0.001 | 5.65  5.47  3.91 | 22  28  26 | -54  -64  -76 | 10  2  -10 |
| Postcentral Gyrus (BA 6)  Precentral Gyrus (BA 6) | L  L | 145 | P<0.001 | 6.05  5.28 | -48  -36 | -12  -10 | 54  52 |
| SMA (BA 6)  SMA (BA 6) | L  R | 112 | P=0.002 | 6.03  4.81 | -10  6 | 12  10 | 48  46 |
| Middle Frontal Gyrus (BA 46)  Inferior Frontal Gyrus (BA 47) | R  R | 93 | P=0.002 | 5.90  5.80 | 36  38 | 40  36 | 12  10 |
| Inferior Frontal Gyrus (BA 44) | R | 79 | P=0.008 | 6.03 | -44 | 6 | 24 |
| Middle Temporal Gyrus | R | 70 | P=0.013 | 5.48 | 54 | -42 | 2 |
| Postcentral Gyrus (BA 3) | L | 65 | P=0.017 | 5.71 | -44 | -16 | 32 |
| Precentral Gyrus (BA 4) | R | 60 | P=0.022 | 5.38 | 50 | -12 | 40 |
| Amygdala (LB) | L | 39 | P=0.042 | 6.35 | -32 | -2 | -24 |
| Thalamus | R | 38 | P=0.048 | 5.20 | 18 | -14 | 8 |
