# Supplementary material for: Brain Circuitries Involved in Semantic Interference by Demands of Emotional and Non-Emotional Distractors
Source: PLoS One. 2012 May 29;7(5):e38155. doi: 10.1371/journal.pone.0038155 (PMC3362560; doi:10.1371/journal.pone.0038155)
Supplement: Table S4 — Areas that positively correlated with conflict-related slowing during incongruent trials in the non-emotional task at P<0.05 cluster-level family-wise error-corrected; cluster-forming threshold at voxel-level P<0.0001. (DOC) [file pone.0038155.s004.doc]

**Table S4**

**Areas that positively correlated with conflict-related slowing during incongruent trials in the non-emotional task at P<0.05 cluster-level family-wise error-corrected; cluster-forming threshold at voxel-level P<0.0001**

| **Anatomical region** | **Side** | **k** | **FWE-corrected**  **corrected** | **Peak voxel** | | | |
| --- | --- | --- | --- | --- | --- | --- | --- |
|  |  |  |  | **T** | **x** | **y** | **z** |
| Inferior Frontal Gyrus (BA 44) | L | 58 | P=0.019 | 6.23 | -44 | 26 | 24 |
| Inferior Parietal Lobule | L | 50 | P=0.031 | 6.23 | -50 | -20 | 36 |
